# Autonomous Non Antioxidant Roles for *Fasciola hepatica* Secreted Thioredoxin-1 and Peroxiredoxin-1

**DOI:** 10.3389/fcimb.2021.667272

**Published:** 2021-05-05

**Authors:** Amber Dorey, Krystyna Cwiklinski, James Rooney, Carolina De Marco Verissimo, Jesús López Corrales, Heather Jewhurst, Barbara Fazekas, Nichola Eliza Davies Calvani, Siobhán Hamon, Siobhán Gaughan, John P. Dalton, Richard Lalor

**Affiliations:** Molecular Parasitology Laboratory, Centre of One Health (COH), Ryan Institute, National University of Ireland, Galway, Ireland

**Keywords:** *Fasciola*, helminth, antioxidants, thioredoxin, thioredoxin peroxidase, peroxiredoxin, immunomodulation, inflammation****

## Abstract

Trematode parasites of the genus *Fasciola* are the cause of liver fluke disease (fasciolosis) in humans and their livestock. Infection of the host involves invasion through the intestinal wall followed by migration in the liver that results in extensive damage, before the parasite settles as a mature egg-laying adult in the bile ducts. Genomic and transcriptomic studies revealed that increased metabolic stress during the rapid growth and development of *F. hepatica* is balanced with the up-regulation of the thiol-independent antioxidant system. In this cascade system thioredoxin/glutathione reductase (TGR) reduces thioredoxin (Trx), which then reduces and activates peroxiredoxin (Prx), whose major function is to protect cells against the damaging hydrogen peroxide free radicals. *F. hepatica* expresses a single TGR, three Trx and three Prx genes; however, the transcriptional expression of Trx1 and Prx1 far out-weighs (>50-fold) other members of their family, and both are major components of the parasite secretome. While Prx1 possesses a leader signal peptide that directs its secretion through the classical pathway and explains why this enzyme is found freely soluble in the secretome, Trx1 lacks a leader peptide and is secreted *via* an alternative pathway that packages the majority of this enzyme into extracellular vesicles (EVs). Here we propose that *F. hepatica* Prx1 and Trx1 do not function as part of the parasite’s stress-inducible thiol-dependant cascade, but play autonomous roles in defence against the general anti-pathogen oxidative burst by innate immune cells, in the modulation of host immune responses and regulation of inflammation.

## Introduction

Digenean trematodes are internal obligate parasites responsible for a plethora of foodborne zoonotic diseases in humans and their livestock. They have a complex life cycle that involves migration within multiple different intermediate and definitive host species. They can reside within their definitive mammalian host for years, and even decades. They include the liver flukes (*Fasciola* spp., *Opisthorchis* spp., and *Clonorchis* spp.), blood flukes (*Schistosoma* spp.) and lung flukes (*Paragonimus* spp.) that, collectively, infect over 250 million people worldwide (Keiser and Utzinger, 2009; Furst et al., 2012).

Fasciolosis caused by infection with *Fasciola hepatica* is classically associated with livestock (sheep and cattle) on farms in temperate climates. Due to human migration and animal trade over the past few centuries the disease has one of the most widespread geographical distributions of any helminth (Robinson and Dalton, 2009). The spread of the disease has been enhanced by the superior adaptability of this parasite to its different hosts since it can infect, develop and produce off-spring in many mammals that it has only encountered in relatively recent times e.g., camelids, capybara and kangaroos. Fasciolosis caused by *Fasciola gigantica*, on the other hand, is most prevalent in tropical regions where it is most commonly found in cattle and water buffalo (Copeman and Copland, 2008; Mas-Coma et al., 2019). Where both *F. hepatica* and *F. gigantica* are sympatric, for example in China, Korea, and Southeast Asia, hybrids forms of the parasite have emerged (Calvani and Šlapeta, 2021).

Both *F. hepatica* and *F. gigantica* have a similar life cycle involving an intermediate snail and definitive mammalian host. The mammalian hosts become infected after they consume encysted parasites (metacercariae) attached to vegetation (grass, rice) or floating in water (Andrews, 1999). The metacercariae emerge from their cysts as newly excysted juveniles (NEJs) in the low-oxygen environment of the small intestine and, with the assistance of abundant protease secretion, traverse the intestinal wall within hours (Andrews, 1999; Cwiklinski et al., 2018). Aside from small tracks, the microscopic *F. hepatica* NEJs leave little clinical evidence of their travels through the intestinal wall that lack any signs of immune cellular infiltration to the vicinity of challenge in naïve animals (Van Milligen et al., 1998).

Serious damage begins after the parasite enters the liver and begins migration through the parenchymal tissues, again with the aid of secreted proteases, causing excessive haemorrhaging, which results in anaemia (Molina-Hernández et al., 2015). It is this damage that results in poor animal growth and loss of productivity (wool, meat and milk yields), the extent of which depends on the level of infection where large numbers of *F. hepatica* entering the liver around the same time can cause sudden death in sheep (Molina-Hernández et al., 2015; Nadis, 2020). After about 8-12 weeks *F. hepatica* migrates into the bile ducts where it matures and uses the nutrients from its obligate blood feeding activity to produce numerous progeny in the form of eggs. Eggs are passed in faeces, where they eventually embryonate on pasture before hatching to release miracidia that go on to infect the intermediate snail host. Within the snail host the parasites multiply *via* clonal expansion before emerging as cercariae that encyst as metacercariae on vegetation contaminating pastures, thus continuing the life cycle (Graczyk and Fried, 1999; Hodgkinson et al., 2018).

Similar to that observed in other helminth infections, the early immune response to the invading parasite is mixed or non-polarised (Espino and Rivera, 2009). However, within a week of infection the developing immune response in mice exhibits all the hallmarks of a strongly polarised Th2-driven response; including, the dominance of IgG1 antibody isotypes over IgG2 (Phiri et al., 2006), the recruitment and proliferation of eosinophils (Ruiz-Campillo et al., 2017), the differentiation of alternatively activated macrophages (M2s) (Donnelly et al., 2008), the secretion of IL-4/IL-5/IL-13 by T-cells (Espino and Rivera, 2009), as well as the suppression of Th1-associate cytokines (O’Neill et al., 2000). Studies in ruminants suggest that sheep and cattle also elicit Th2-driven responses to acute infection, which progresses into a hyporesponsive or immunosuppressive state as the disease becomes chronic (Escamilla et al., 2016; Sachdev et al., 2017). Several studies suggest that Th2-driven immune responses make the host susceptible to subsequent infection (Aitken et al., 1981; Chauvin et al., 1995; Brady et al., 1999; Cwiklinski et al., 2016), and both infection and vaccine studies suggest that it is necessary to induce Th1-mediated responses for protection to be achieved (Pleasance et al., 2011; Villa-Mancera et al., 2014; Noya et al., 2017).

## The Best Form of Defence Is Having a Good Offence

The ability of *F. hepatica* to survive and thrive in its varied mammalian hosts for such a long time is reflective of the parasites’ capacity to evade, modulate or supress the host’s immune responses (Molina-Hernández et al., 2015). The parasites immune evasion techniques include the continual sloughing of their exterior ‘fuzzy’ surface glycocalyx, along with bound host antibody, thus rendering it ineffective (Hanna, 1980a; Lammas and Duffus, 1983; Haçarız et al., 2011). Additionally, the antigenic and structural composition of the glycocalyx changes as the parasites migrate from intestine to liver to bile duct, leaving the successively-mounting host immune responses redundant (Hanna, 1980b). Histological observations of livers taken from infected hosts show that the damage caused by the aggressive and rapid tunnelling of the parasite becomes infiltrated with an immense amount of immune cells (eosinophils, lymphocytes, macrophages). However, rather than killing the parasite these cells appear to be playing the role of plugging the tracts left in the parasites’ wake, preventing excessive blood loss and, most importantly, facilitating wound repair as the tracts gradually become fibrotic and sealed with collagen (Zafra et al., 2013a; Zafra et al., 2013b; Frigerio et al., 2020).

The best form of defence, however, is having a good offence, and helminth parasites achieve this largely by the excretion/secretion of a multitude of immune-impairing, -suppressive or -modulatory factors (Hewitson et al., 2009; Ryan et al., 2020). In the case of *F. hepatica*, these include proteases, protease inhibitors, antioxidants, cathelicidin-like helminth defence molecules (HDM) and glycolytic enzymes (Jefferies et al., 2001; Morphew et al., 2007; Ryan et al., 2020), many of which have been shown to influence different aspects of the host’s immune response. For example, early studies showed that secreted cysteine proteases (now known as cathepsin B and cathepsin L proteases) can specifically cleave immunoglobulins (Igs) at their hinge region, separating the antibody binding Fab fragment from the Fc domain and thus preventing the ability of bound Ig to attract Fc-binding innate immune cells (eosinophils, macrophages) (Chapman and Mitchell, 1982). The secreted *F. hepatica* HDMs abrogate NLP3-inflammasome-mediated inflammatory responses in innate immune cells by impairing lysosomal acidification (Robinson et al., 2012; Alvarado et al., 2017), while fatty acid binding proteins (FABP 12/15) are shown to induce alternatively activated macrophages that overexpress anti-inflammatory cytokines, thereby contributing to a hyporesponsive environment favoured by the parasite (Figueroa-Santiago and Espino, 2014; Ruiz-Jiménez and Espino, 2014; Ramos-Benítez et al., 2017). Several molecules, such as *F. hepatica* cathepsin L1 cysteine proteases, glutathione S- transferases (GST) and Kunitz-type molecules, reduce the capacity of dendritic cells to induce the robust T-cell responses required to effectively eliminate the parasite (Dowling et al., 2010; Falcon et al., 2014; Ryan et al., 2020). Glycosylated mucins and TGF-β mimics secreted by invading parasites may also play immunosuppressive or immunoregulatory roles by influencing DC or T-cell phenotype differentiation to block Th1 type responses developing (Musah-Eroje and Flynn, 2018). Metabolism-associated enzymatic factors liberated by the parasite and not classically associated with immunosuppression, such as fructose-bisphosphate aldolase and glyceraldehyde phosphate dehydrogenase, have recently been shown to bind to the host’s immune mediating factors such as IFN-γ, IL-2 and IL-17 (Liu et al., 2017). However, the exact effect that the binding of *F. hepatica* products has on these factors remains to be elucidated.

Detailed genomic, transcriptomic, and proteomic (somatic and secretome) analyses that have emerged over the last few years have revealed that the liver fluke parasite tightly regulates the expression and secretion of many molecules during its migration in the mammalian host (Cwiklinski and Dalton, 2018). The growth from a microscopic organism to a mature egg-producing adult parasite (~2 cm x 1 cm) is correlated with the up-regulation and differential expression of a range of gene families critical for the stage-specific phases within the mammalian host (Cwiklinski et al., 2015). In particular, an increased transcription of >8000 transcripts, many of which encode pathways involved with intense signal transduction, protein production and neoblast development, is observed when the parasite invades and migrates through the liver parenchyma (Cwiklinski et al., 2021). Direct associations can also be made between transcript up-regulation and the secretion of molecules involved in the parasite-host inter-relationship e.g., the aforementioned secreted immune regulatory proteases, protease inhibitors and HDMs (Robinson et al., 2009; Cwiklinski et al., 2018; Cwiklinski et al., 2021).

**Figure 1:**
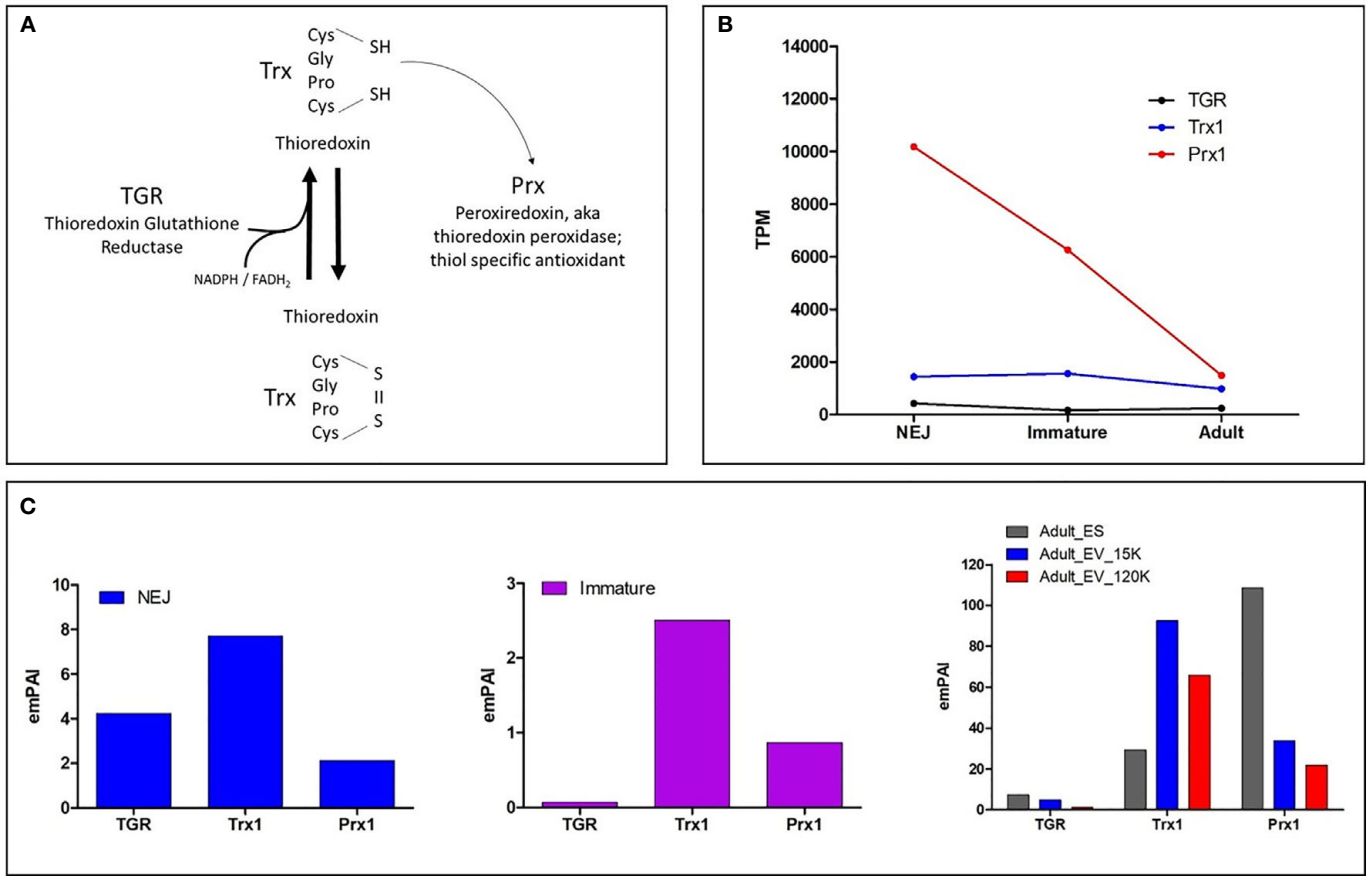
**(A)** Schematic of the generalised model of the thiol-dependent antioxidant cascade. TGR with the assistance of FADH_2_ and NADPH converts oxidised Trx to reduced Trx which subsequently reduces Prx to its activated form. **(B)** Graphical representation of the most abundantly transcribed genes of the three enzymes in the *F. hepatica* thiol-dependent antioxidant cascade by the newly excysted juveniles (NEJ) that traverse the small intestinal wall, the liver stage immature parasites 21 days post infection (Immature) and the mature adult stage parasite that resides within the bile ducts (Adult). Data is displayed as transcripts per million (TPM) and is extrapolated from the transcriptome study by Cwiklinski et al. (2015). **(C)** Graphical representation of the protein abundance within the NEJ, Immature and Adult parasite secretomes (ES proteins), represented by Exponentially Modified Protein Abundance Index (emPAI). The Adult secretome data shows the protein abundance within the extracellular vesicles, specifically the microvesicles recovered following centrifugation at 15, 000 x g (Adult_EV_15K) and the exosomes isolated after centrifugation at 120, 000 x g (Adult_EV_120K), in addition to the EV-depleted ES proteins. The proteomic data for NEJ, immature and adult parasites is extrapolated from Cwiklinski et al. (2018), Cwiklinski et al. (2021) and Murphy et al. (2020), respectively.

An interesting observation emerged when the expression of various antioxidant enzymes of the migrating parasite were examined alongside the expression of homologous enzymes in the host liver, suggesting that metabolic stress occurs in both the parasite and liver (Cwiklinski et al., 2021). The mammalian liver is naturally a high-metabolising organ susceptible to oxidative stress in many infectious and non-infectious chronic diseases, including hepatitis C, alcoholic liver disease and liver fibroproliferative disease (Cichoż-Lach and Michalak, 2014). The elevation of metabolic stress in the liver during infection with *F. hepatica* is therefore not surprising since a major effort is required to recruit immune cells to damaged areas in order to minimise necrosis, repair perforated tissue and to induce fibrogenesis (Saleh, 2008; Bottari et al., 2015; Da Silva et al., 2017). In contrast, the metabolic stress exerted on the parasite arises from two sources; (1) internally by the increased cellular metabolic activity and associated generation of reactive oxygen species (ROS) driven primarily as a result of the aerobic respiration by the rapidly growing and developing parasite, and (2) externally by the parasite’s need to respond to the general anti-pathogen ROS burst from the host’s innate immune cells. Both of these metabolic pressures demand the up-regulation of transcripts encoding superoxide dismutase (SOD), glutathione peroxidase (GPx), glutathione S-transferase (GST), as well as members of the thiol-dependent antioxidant system (**Figure 1A**). The thiol-dependent antioxidant system is the primary means by which cells and parasites defend against the major biological ROS hydrogen peroxide (H_2_O_2_). Recent studies have, however, shown that various players in this system are not only part of a defence mechanism but may also perform a range of functions that can be central to the parasite’s ability to manipulate the host immune responses (Ishii et al., 2012; Perkins et al., 2014).

## Enzymes in the *F. hepatica* Thiol-Dependent Antioxidant Cascade

The *F. hepatica* thiol-dependent antioxidant cascade includes the enzymes thioredoxin-glutathione reductase (TGR), thioredoxin (Trx) and thioredoxin peroxidase/peroxiredoxin (Prx). These proteins interact *via* a redox cascade event whereby TGR reduces Trx with the assistance of nicotinamide adenine dinucleotide phosphate (NADPH), which in turn reduces Prx to recharge its redox state and activates its antioxidant properties (**Figure 1A**).

TGR, the first protein involved in this redox antioxidant cascade, is an oxidoreductase enzyme that can reduce both Trx and glutathione disulfide (GSSG) in a 1:1 ratio, in much the same way as thioredoxin reductase (TrxR) and glutathione reductase (GR) separately perform this function in mammalian cells. It is important to note, however, that no independent TrxR or GR enzymes have been identified in *F. hepatica* (Guevara-Flores et al., 2011). Sequence analysis of TGR has identified binding domains suitable for both NADPH and FAD, a thiol-disulphide redox active centre that has been described for mammalian TR and GR, as well as a glutaredoxin (Grx) domain (Maggioli et al., 2011). TGR enzymes are atypical due to the presence of a selenocysteine insertion sequence (SECIS) element, encoded by a TGA codon that enables the incorporation of selenium into the TGR protein (Böck et al., 1991). A study of the structure of *F. gigantica* TGR demonstrated the requirement for the selenocysteine (Sec) element for both TrxR and GR activity (Kalita et al., 2018). The enzymes’ high sensitivity to inhibition by aurothioglucose confirmed it as a selenoenzyme (Maggioli et al., 2004). The inhibition of *Schistosoma mansoni* TGR by auroanofin, an antirheumatic compound (Kuntz et al., 2007), is facilitated by the binding of the compound between the catalytic cysteines of the FAD-binding site (Cys154-Cys159), preventing the donation of electrons to the enzyme by flavin adenine dinucleotide (FAD) (Angelucci et al., 2009). Similar investigations into the inhibition of *F. gigantica* TGR by auranofin determined that it is the interaction of the gold particle of auranofin with His571 of TGR that results in the inhibition of enzyme activity (Kalita et al., 2018). The *S. mansoni* TGR was proposed as a tractable drug target due to the parasites’ inability to survive in the presence of auranofin (Feng et al., 2020).

Trx is a ~12 kDa oxidoreductase protein with a catalytically active dithiol site that reduces exposed disulfide bridges on Prx and other proteins. It has a conserved structure composed of a core that is formed from a four-stranded β-sheet, surrounded by three α-helices (Eklund et al., 1984; Martin, 1995). The tryptophan-cysteine-glycine-proline-cysteine (WCGPC) motif of the Trx active site protrudes from the 3-D structure of the molecule and is highly conserved (Shoda et al., 1999). The redox active cysteine pair (Cys31 and Cys34) (Ren et al., 2017) enables the enzyme to exist in either the oxidised disulphide state or the reduced dithiol state (Line et al., 2008). A proline situated between the two cysteines (Pro33) is essential to facilitate the reducing power of Trx (Collet and Messens, 2010). The substitution of this proline with histidine in *Escherichia coli* Trx resulted in a lower reducing potential compared to the wild-type enzyme (Krause et al., 1991). Similarly, in *Staphylococcus aureus* the substitution of the proline with either serine or threonine resulted in a seven-fold reduction in the reducing potential of the enzyme (Roos et al., 2007).

Prxs are found in both prokaryotes and eukaryotes and are well characterised in many protists and helminth parasites (see review by Angelucci et al., 2016). Their discovery in *F. hepatica* helped explain how helminth parasites deal with SOD-generated hydrogen peroxide since they lack the enzyme catalase that together with glutathione peroxidase (GPx) converts the toxic reactive oxygen molecules into water and hydrogen peroxide (McGonigle et al., 1997; McGonigle et al., 1998). Since the discovery of Prx, the enzyme has undergone several name changes, first described as thiol specific antioxidants and then thioredoxin peroxidase (which often still appears in helminth proteomic studies) before being termed peroxiredoxin (**Figure 1**). Prx is a 25 kDa enzyme that when activated to its reduced form by Trx, provides protection to the parasite *via* the breakdown of hydrogen peroxide (McGonigle et al., 1997), a mechanism reliant upon a conserved cysteine residue in the enzyme’s NH_2_-terminal portion (Rhee et al., 2001). The enzyme found in *F. hepatica* shows high levels of homology to other peroxiredoxin enzymes, including those found in rodents, ruminants, and humans (Salazar-Calderón et al., 2000). The peroxiredoxin of *F. hepatica* is a 2-Cys enzyme, the most widely distributed subfamily of Prxs (Hall et al., 2009), that is characterised by two active cysteine residues at positions 47 and 170 (McGonigle et al., 1997). Incubation of recombinant *F. hepatica* peroxiredoxin, rFhePrx, with super-coiled plasmid DNA and DTT demonstrated the ability of the antioxidant to protect against oxidative stress (Sekiya et al., 2006), and supports the idea that *F. hepatica* produces Prx in order to protect itself from oxidative damage within the host environment. It has been shown that Prxs can form dimers and higher molecular size multimers depending on the redox status of the cell; at low oxidative stress they can act as peroxidases whereas at high levels of stress they act as holdases, enzymes that can assist the non-covalent folding of proteins and prevent protein aggregation (Jang et al., 2004; Teixeira et al., 2015). This interchange of oligomeric states has been shown for the 2-Cys peroxiredoxins of adult *S. mansoni* and provides an explanation of how a sensing mechanism for hydrogen peroxide concentration can be translated to a functional molecular switch (Saccoccia et al., 2012). At present, we can only assume a similar mechanism exists for *F. hepatica* Prx.

## What Does Our -Omics Analysis Reveal About the Enzymes in the *F. hepatica* Thiol-Dependent Antioxidant Cascade?

Our recent analysis of the *F. hepatica* genome published by Cwiklinski et al. (2015) revealed that TGR is encoded by a single copy gene, and transcriptomic analysis shows this is constitutively expressed during the stages that infect the mammalian host. The genome contains three genes that encode Trx enzymes, Trx1, 2 and 3, all of which are also constitutively expressed. However, Trx1 is expressed >50 times higher than both Trx2 and 3 (each less than 40 transcripts per million; TPM) (**Figure 1B**). There are also three Prx genes present within the *F. hepatica* genome (Prx1, 2 and 3) but, as in the case for the Trxs, the expression of one, Prx1, greatly outweighs the other two genes. Prx1 displays stage-specific transcription, with the highest expression observed during the NEJ stage at >250 times greater (~10,000 TPM) than the other two Prx genes and the other members of the thiol-dependent antioxidant cascade (**Figure 1B**). This disparity in the expression of Trx1 and Prx1 sets them apart from the other members of their family, implying that they play alternative roles.

Proteomic profiling of the secreted proteins of the infectious NEJs, immature 21-day post-infection and adult parasites identified TGR, Trx1 and Prx1 but no other members of the thiol-dependent antioxidant cascade (Cwiklinski et al., 2018; Murphy et al., 2020; Cwiklinski et al., 2021) (**Figure 1C**). TGR was detected in the NEJ secretome, but was only minimal in that of the immature and mature adult parasite. In contrast, both Trx1 and Prx1 were abundant in all secretomes, although Trx1 was more dominant in the NEJ stage while, conversely, Prx1 was most abundant in the adult parasite secreted products. We also found Trx1 and Prx1 enzymes within the contents of different sized extracellular vesicles/exosomes recovered by differential centrifugation (15K and 120K; Murphy et al., 2020) from adult worm secretory products. The majority of Trx1 observed was associated with the microvesicles (15K) while Prx1, although present within both the EV fractions, was predominantly in the soluble non-vesicle fraction and thus it seems unlikely that the two interact in a reducing cascade.

## Leader (LP) and Leaderless (LLP) Protein Secretory Pathways: Trx1 and Prx1 go Their Separate Ways

Since Trx1 and Prx1 are found in abundance in the secretory products of *F. hepatica*, it follows that they are readily released into the extracellular environment by the parasite and, by extension, likely to be released *in vivo* during infection (Murphy et al., 2020). Conventionally in eukaryotic organisms, proteins destined for secretion contain an N-terminal hydrophobic signal peptide that targets the protein for translocation and processing in the endoplasmic reticulum and Golgi apparatus. The proteins are subsequently packaged into secretory vesicles that fuse to the plasma membrane and release the proteins freely into the extracellular environment (Palade, 1966; Blobel and Dobberstein, 1975). Our recent analysis of the *F. hepatica* genome discovered that Prx1 is unique amongst the three-membered family in possessing a signal secretory peptide suggesting it is secreted *via* the classical leader pathway (LP). This would explain its predominance in the secretome and, more relevantly, in the freely-soluble fraction.

In contrast, Trx1 falls into the general category of leaderless secretory proteins (LLPs), of which the mechanism of cellular release remains less clearly understood but may occur through numerous unconventional processes (Sitia and Rubartelli, 2020). Direct translocation of LLPs through the plasma membrane *via* lipidic or proteinaceous pores is one proposed mechanism (Steringer et al., 2012; He et al., 2015). Packaging of these proteins into autophagosomes, multivesicular bodies, and secretory endolysosomes is another established mechanism involved in the translocation of LLPs across the plasma membrane (Dupont et al., 2011; Zhang et al., 2015). It is clear from our studies that Trx1 is packaged into extracellular vesicles/exosomes before release from the surface tegument or from gastrodermal epithelial cells. Therefore, Trx1 is unlikely to be freely soluble but more probably delivered to host cells along with the total exosome cargo (de la Torre-Escudero et al., 2019; Murphy et al., 2020).

## Prx1 and Trx1 – Specialised Immunomodulatory Proteins?

The results of our analysis of the *F. hepatica* genome is in keeping with the idea that a functional thiol-dependent antioxidant cascade operates as a defence system against metabolic stress in this parasite. However, it is unclear how TGR, which is encoded by a single copy gene, interacts with the triple Trx and Prx members of this system. Perhaps the various Trxs and Prxs are expressed in different parasite tissues that are under varying levels of metabolic stress (e.g., tegument, reproductive system, gastrodermis etc) and/or duplication of the anti-oxidant genes have generated enzymes with enhanced or varied functions, a feature we have observed in the expanded families of cysteine proteases and protease inhibitors (Cwiklinski et al., 2019; De Marco Verissimo et al., 2020; Smith et al., 2020). Obviously, elucidation of this conundrum awaits more detailed biochemical and cellular studies of each antioxidant member. Notwithstanding, the abundant gene expression and secretion of Trx1 and Prx1 is at variance with a role for these two anti-oxidants alongside the other members in the general cellular metabolism of the parasite and is more in line with their involvement in specialised host-parasite interactions i.e., direct manipulation of host responses. Furthermore, the distinct secretory routes taken by Trx1 and Prx1 would suggest that these are not functional partners but act autonomously. So, what could the function of Trx1 and Prx1 be?

A secondary role for Prx, which we now know is Prx1, in host immune modulation was previously described by us after the antioxidant was discovered as a major component of a fraction of adult *F. hepatica* ES products that induced Th2-immune responses in mice (Donnelly et al., 2005; Donnelly et al., 2008). We subsequently found that addition of a functionally-active recombinant form of Prx1 to cultured macrophages induced their differentiation into M2s. Moreover, intraperitoneal injection of BALB/c mice with the same protein induced the recruitment into the peritoneal space of M2s that were not responsive to stimulation with LPS (Donnelly et al., 2008). Since M2s play a key role in maintaining the Th2 responses of the host immune system, as well as in suppressing the host inflammatory response (Donnelly et al., 2008), we proposed a role for Prx1 in the immunoregulation of the host response by *F. hepatica*. Importantly, the ability of Prx1 to induce M2s *in vitro* and *in vivo* was independent of its antioxidant properties since an inactive recombinant variant was equally immunoregulatory as the wild-type enzyme. We also demonstrated that host (mouse) Prx had similar M2 properties when injected intraperitoneally, prompting us to suggest that parasite Prx acts like a host damage-associated molecular pattern (DAMP).

Unlike Prx1, however, novel functions of *F. hepatica* Trx remain to be discovered. Sequence alignments and structural models of the various *F. hepatica* Trxs with those from other parasites (protozoan and helminth) and mammals reveal a fully conserved structure including the protruding five amino acid motif (WCGPC) at its active site and neighbouring residues vital for its functionality (Salazar-Calderón et al., 2001; Changklungmoa et al., 2014). This could infer that FhTrx1 exhibits some or all of the expanding assortment of endogenous and exogenous activities that are emerging for human Trx1 (hTrx). For example, besides reducing Prx intracellularly, Trx acts as a hydrogen donor for proteins involved in DNA synthesis (Zahedi Avval and Holmgren, 2009), is involved in the redox control of the inflammatory related transcription factors like NF-κB and AP-1 (Schenk et al., 1994), and prevents apoptosis *via* direct binding with apoptosis signal-regulating kinase (Liu and Min, 2002). Its extracellular activity is predominantly mediated enzymatically rather than through classical receptor-like binding (Bertini et al., 1999) and includes reduction and modulation of the activity of extracellular receptors (Schwertassek et al., 2007; Xu et al., 2008) and reduction of IL-1beta mRNA and protein synthesis through suppression of NF-kB activation (Billiet et al., 2005). Trx maintains extracellular cysteine in its reduced form, which is essential for the survival and expansion of activated T-cells (Angelini et al., 2002), and significantly enhances the production of IL-2 and IL-10 (Sido et al., 2005). Administration of recombinant hTrx abrogated the inflammatory progression of chronic pancreatitis, mediated in part by the inactivation of IL-4 *in vivo* (Plugis et al., 2018). It also reduced acute skin inflammatory reactions and lipopolysaccharide-induced infiltration by desensitising innate immune cells to the chemokines KC, RANTES and MCP-1 (Nakamura et al., 2001; Pagliei et al., 2002; Ono et al., 2013).

## Concluding Remarks

The growth and development of *F. hepatica* in the mammalian host places a major metabolic burden on the parasite as it migrates through host tissues, especially during the early invasive stages that rely on stored glycogen for energy (Bennett and Threadgold, 1973; Bennett, 1977). It is a vulnerable time for the parasites and, therefore, they must possess an effective antioxidant system to protect cells from stress-related oxidative damage. The parasites must contend with ROS-mediated attack from the host immune effector cells, such as macrophages and eosinophils, and a rapidly developing host inflammatory response. Turnover and antigenic changes to the surface tegument are effective mechanisms of immune avoidance but the secretion of molecules is a proactive way in which the parasite can penetrate and feed on its host, as well as manipulate its immune response to assist its survival.

The thiol-dependent antioxidant cascade is an important system for *F. hepatica* to cope with increasing metabolic-derived ROS, but expansion of members of this system by gene duplication has freed up particular members, Trx1 and Prx1, to diverge in function and become part of the parasites’ armoury in defence and, indeed, offense. The absolute function(s) of the secreted Trx1 and Prx1 remain uncertain but their abundance in parasite secretions and our few studies on their activity on immune cells encourage further studies on their potential in immune regulation. Recent studies in mammalian systems are unveiling a diverse range of novel functions for Trx and Prx independent of their antioxidants properties, particularly in the regulation of inflammation (Ishii et al., 2012; Perkins et al., 2014). Indeed, it is tempting to suggest that parasites are intervening in the host immune regulation by molecular and functional mimicry.

Our current -omics derived information provides a sound base to better understand the components of the *F. hepatica* thiol-dependent antioxidant cascade, and indeed other non-thiol dependent antioxidant systems, and their role in parasite physiology and parasite-host interaction. The pivotal role that these systems play in parasite survival makes them tractable targets to which anti-parasite drugs or vaccines could be targeted. Comparative studies by Piedrafita et al. (2000) on the susceptibility of *F. hepatica* and *F. gigantica* NEJs to killing by pro-inflammatory macrophages found a link between antioxidant expression and resistance. More recent studies have demonstrated the effectiveness of the TGR inhibitor gold(I) drug auranofin against several parasites, including schistosomes (Feng et al., 2020), that augurs well for the treatment of *F. hepatica* infection given that the single enzyme is pivotal to the whole cascade (**Figure 1**). The Trx1 and Prx1 enzymes may also be considered targets for vaccine-induced immune responses, either alone, together or in a cocktail with other antioxidants.

## Author Contributions

All authors listed have made a substantial, direct, and intellectual contribution to the work and approved it for publication.

## Funding

This work was funded by the Science Foundation Ireland (SFI, Ireland) Research Professorship grant 17/RP/5368.

## Conflict of Interest

The authors declare that the research was conducted in the absence of any commercial or financial relationships that could be construed as a potential conflict of interest.
